# A Novel Human TPIP Splice-Variant (TPIP-C2) mRNA, Expressed in Human and Mouse Tissues, Strongly Inhibits Cell Growth in HeLa Cells

**DOI:** 10.1371/journal.pone.0028433

**Published:** 2011-12-02

**Authors:** Rasmi Rekha Mishra, Jitendra Kumar Chaudhary, Gagan Deep Bajaj, Pramod C. Rath

**Affiliations:** Molecular Biology Laboratory, School of Life Sciences, Jawaharlal Nehru University, New Delhi, India; Johns Hopkins School of Medicine, United States of America

## Abstract

Alternative splicing of mRNAs is known to involve a major regulation of gene expression at RNA level in mammalian cells. The PTEN (Phosphatase and TENsin homologue deleted from the human chromosome 10), TPTE (Transmembrane Phosphatase with TEnsin homology) and TPIP (TPTE and PTEN homologous Inositol lipid Phosphatase) belong to a family of dual-specific lipid and protein phosphatases. PTEN is a well characterized tumor suppressor, which plays crucial role in cell survival, cell cycle regulation, cell proliferation as well as adhesion, motility and migration of cells. The C2-domain of PTEN is essential for PTEN-functions. We have isolated a novel 1019 bp human TPIP cDNA (TPIP-C2) from a human testis cDNA library. *In silico* analysis of the cDNA revealed that it is produced from the TPIP-locus on the human chromosome 13 by alternative RNA-splicing. It has a unique 5′-Alu sequence, a LINE sequence followed by a 582 bp Open Reading Frame (ORF) encoding a 193 aa polypeptide with a partial phosphatase domain and a C2-domain. TPIP-C2 mRNA is expressed in human testis and in mouse tissues. Mouse testis and brain showed higher levels of TPIP-C2 mRNA in comparison to the heart, liver and kidney under normal physiological conditions. TPIP-C2 mRNAs from human and mouse testes show extensive sequence identity. Over-expression of TPIP-C2 cDNA in HeLa cells strongly (up to 85%) inhibited cell growth/proliferation and caused apoptosis in a caspase 3-dependent manner. These findings suggest for the first time that a TPIP splice-variant mRNA with a partial phosphatase domain and a C2-domain is expressed in cells and tissues of human and murine origins under normal physiological conditions. Inhibition of cell growth/proliferation and induction of apoptosis by overexpression of TPIP-C2 mRNA in HeLa cells suggest that it may be involved in negative regulation of cell growth/proliferation.

## Introduction

Mammalian genomes contain relatively less numbers of genes, which encode large numbers of proteins. This is effective by alternative splicing of the primary transcripts to generate splice-variants (SVs) of mRNAs, which code for isoforms of proteins with variable functions. The PTEN/MMAC1 (Mutated in Multiple Advanced Cancers-1)/TEP1 (TEnsin-like Phosphatase-1) was identified as a tumour suppressor gene from the human chromosome 10q23.3 [1]–[3]. It is the second most mutated tumour suppressor after *TP53* and is mutated or deleted in a wide variety of cancers. In addition to genetic mutations, somatic, germ-line and promoter mutations of PTEN are responsible for Cowden syndrome (CS), Bannayan-Riley-Ruvalcaba Syndrome (BRRS), Proteus and proteus-like syndrome etc [4]–[6]. PTEN acts as a dual-specific phosphatase, it dephosphorylates both proteins at tyrosine, serine, and threonine residues and lipid second messengers like phosphatidyl inositol 3, 4, 5-triphosphate [PI(3,4,5)P3], -3, 5-diphosphate [PI(3,5)P2], -3, 4-diphosphate [PI (3,4)P2] and -3-phosphate [PI(3)P] at D3-position, thus antagonizing the PI-3 kinase-AKT mediated cell growth/proliferation signaling pathway [7]–[10]. Crystal structure, deletion and mutation studies of PTEN revealed that the C-terminal C2-domain associates strongly with the N-terminal phosphatase domain to make the catalytic site and even small deletion of the C2-domain removes detectable phosphatase activity [11]–[14]. Evidence from recent literature suggests that the C-terminus of PTEN possesses autoinhibitory function, interfering both the phosphatase and C2-domain and this is accomplished by direct interaction of the tail region with the C2-domain [15]–[17]. It is also reported that PTEN regulates cell migration through its C2-domain, independent of its lipid-phosphatase activity and this activity of C2-domain is controlled by Thr^383^ phosphorylation/dephosphorylation [18]. Similarly, PTEN physically interacts with many proteins, e.g., Thioredoxin-1 (Thx-1), serine/threonine kinase (STK11, also named as LKB1) and p53 by its C2-domain [19]–[21]. Interaction of PTEN with p53 facilitates transactivation of p53 and autoregulation of its own expression and this function is independent of the PTEN-phosphatase function [22]. Thus, C2-domain of PTEN is crucial for its biological function.

Other phosphatases of PTEN-family include TPTE and TPIP. TPTE is a testis-specific gene expressed from the human chromosome 21, while TPIP is expressed from the human chromosome 13. Both TPTE and TPIP have multiple splice-variants i.e., TPTEα, β, γ, δ [23]–[26] and TPIP α, β, γ and δ [25], [26] reported so far. TPTE is a testis-specific phosphatase, while TPIPα is highly expressed in testis and brain and at low levels in stomach and TPIPβ is expressed in the testis. The human TPIPγ, TPTEα, TPTEβ and TPTEγ have four putative transmembrane domains; TPIPα and TPTEδ have three and two putative transmembrane domains, respectively, whereas TPIPβ has no transmembrane domain. TPIPα and γ are localized in the endoplasmic reticulum (ER) and Golgi, respectively, TPIPβ is cytosolic. All TPTE derivatives are restricted to the ER and Golgi, except TPTEδ, which shows a more diffused pattern of expression. TPTE and TPIP proteins are expressed in secondary spermatocytes and/or pre-spermatids. TPIP and TPTE have similar domain-organization. TPIPα is reported to be a lipid-phosphatase like PTEN and has phosphatase activity against the lipid substrates: PI(3,4,5)P3, PI(3,5)P2, PI(3,4)P2 and PI(3)P, which are second messengers in cellular signaling pathways [23]–[26].

In the present study, we have isolated a novel TPIP splice-variant (TPIP-C2) mRNA from a human testis cDNA library. TPIP-C2 mRNA can code for a 193 aa putative C2-domain-like protein. It is produced from the human TPIP-locus on chromosome 13 by alternative RNA-splicing and expressed in human testis and mouse tissues. The TPIP-locus of mouse genome is expressed as TPIP-C2 mRNA in the tissues. Functional assay showed that ectopic expression of TPIP-C2 cDNA caused up to 85% suppression of cell growth/proliferation and induced apoptosis in human cervical carcinoma (HeLa) cells. Therefore, the alternatively spliced TPIP-C2 mRNA encoding an isolated C2-domain-like protein may exist in mammalian cells and tissues and negatively regulate signaling events involved in regulation of cell growth/proliferation.

## Results

### Isolation and characterization of TPIP-C2 cDNA from human testis

**Figure 1:**
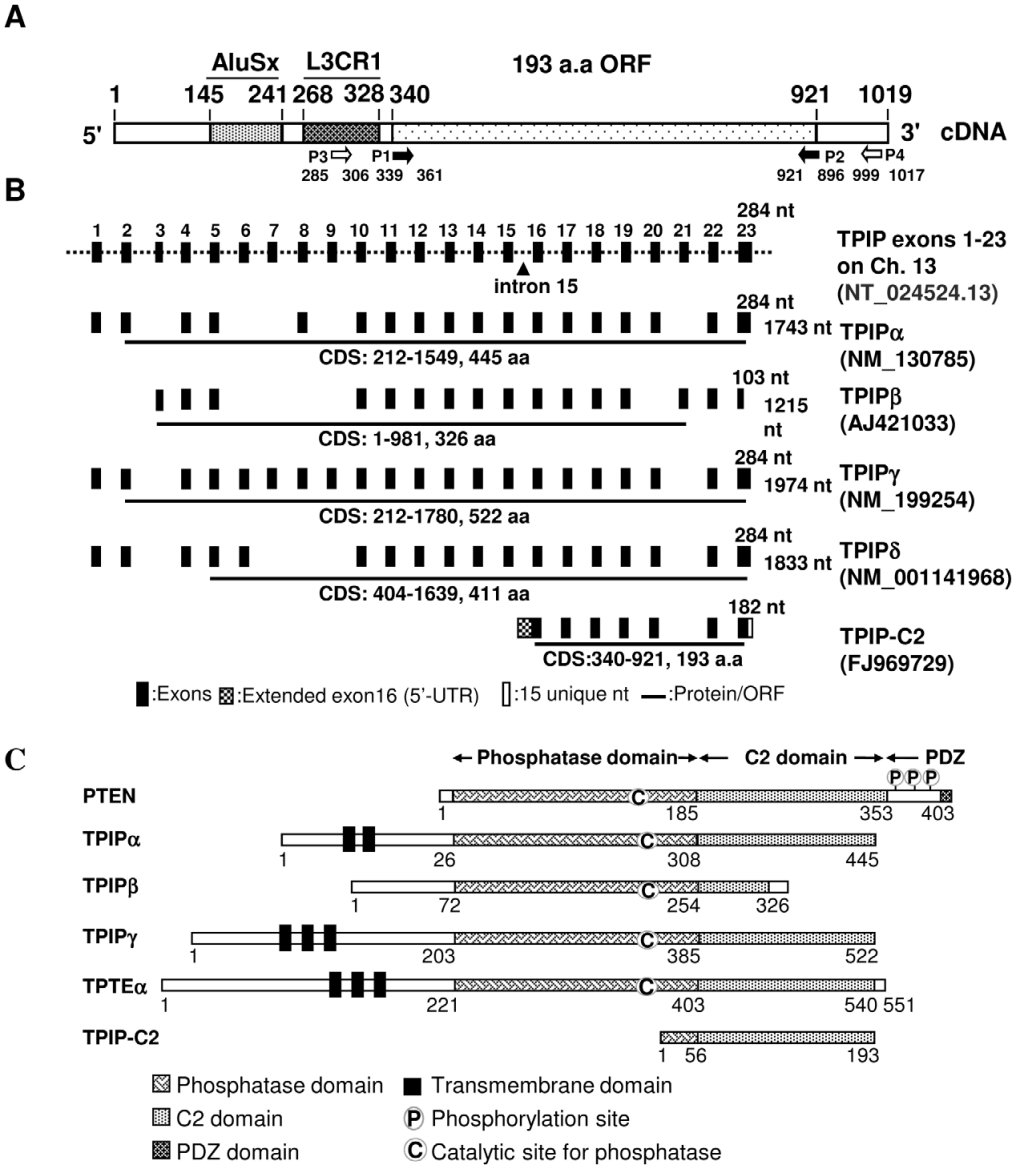
TPIP-C2 cDNA, genomic organization of TPIP-locus and PTEN, TPIP, TPTE protein isoforms. (A) TPIP-C2 cDNA (1019 nt) contains a SINE belonging to the AluSx class and a LINE belonging to the L3CR1 class in its 5′-untranslated region (5′-UTR) followed by a 193 aa. ORF. The P1 and P2 primers amplify a 603 bp TPIP-C2 ORF, the P3 and P4 primers are TPIP-C2-specific and amplify a 733 bp product. (B) Comparison of TPIP-C2 exons with TPIP-isoforms. The 1–23 exons of TPIP gene on the human chromosome 13 and the corresponding exons of TPIP-isoforms are shown. TPIP-C2 cDNA is shown below and the 5′-UTR in shaded box indicates its origin from intron 15. The line below the exons represents CDS and the corresponding ORF, the extended exon 16, the 15 nt unique sequence to exon-23 of TPIP-C2 are shown. (C) Schematic representation of TPIP-C2 and the related protein isoforms. The 193 aa. TPIP-C2 ORF has a partial phosphatase domain (1–56) and a C2-domain (57–193) identical to TPIPα, γ, δ and homologous to PTEN. The structural and functional regions are indicated.

The TPIP-C2 cDNA was isolated as a 1.019 kb cDNA from a λgt11 human testis cDNA library [27] by screening with a 227 bp rat genomic simple repeat DNA probe (GenBank Accession No. X 97459) as described in the materials and methods section during an investigation to look for repeat sequence containing RNAs from the human genome. Analysis of the 1.019 kb TPIP-C2 cDNA sequence (GenBank Accession No. FJ969729) was carried out using standard bioinformatics tools and the summary of the results is schematically shown in Figure 1A, B, C and Figure S1A, B, C. It contains a 5′-97 nt. (145–241) Alu-repetitive SINE element belonging to *AluSx*-class, a 61 nt. (268–328) LINE sequence belonging to L3CR1 class followed by a 582 nt (340–921) encoding a 193 aa ORF (Figure 1A). The genomic organization of TPIP-C2 cDNA was deduced by comparison with the TPIP locus of human genome sequence, as shown in Figure 1B. TPIP-C2 is a novel isoform belonging to PTEN-C2 superfamily, though similar in many ways with the TPIPα, γ and δ isoforms. It has some distinct characteristics, such as presence of a repeat-rich 5′-UTR (46% of the 5′-UTR is composed of SINE and LINE sequences) and a 14 nucleotide unique sequence at 3′-end, which has been incorporated from outside the TPIP-locus (Table S1, Figure 1A, B, C). This may involve trans-splicing, a less frequent RNA-processing mechanism. *In silico* analysis mapped the 5′-end of TPIP-C2 to the intron 15 of the human TPIP locus on chromosome 13 (Figure 1B, Figure S1D). The TPIP-C2 SINE is unique because it is present in the 5′-untranslated region (5′-UTR). In contrast, similar SINE elements are present in 3′-UTRs of several human transcripts (Table S2). It is likely that it may be a target of small non-coding RNA.

**Figure 2:**
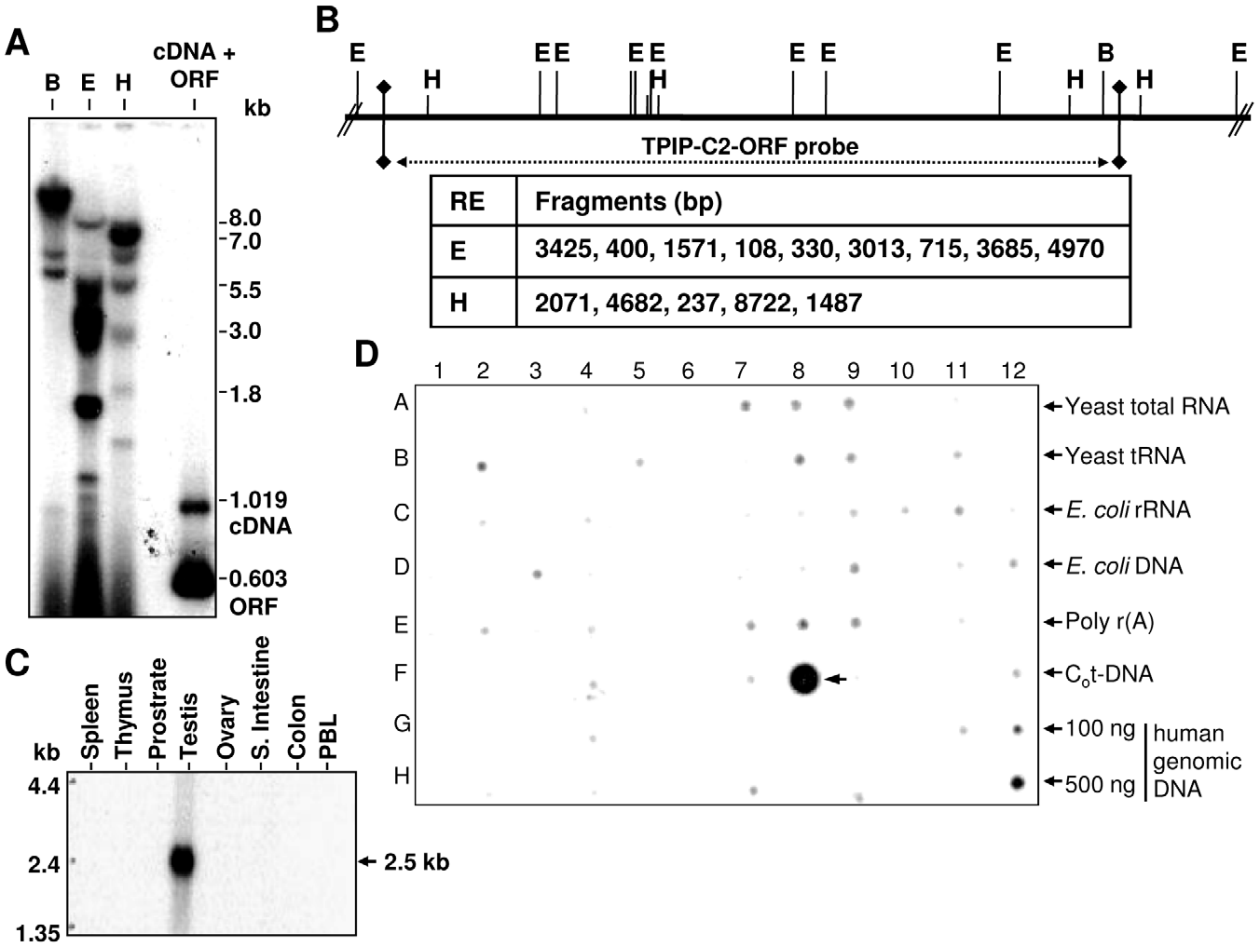
Genomic organization and expression of TPIP-C2 mRNA. (A) Southern blot analysis of genomic DNA from human peripheral blood lymphocytes (PBL) is shown after BamH I (B), EcoR I (E), Hind III (H) digestions and hybridization with [^32^P] labeled 603 bp TPIP-C2 ORF-DNA probe. The cDNA plus ORF is used as a positive control. (B) Restriction map of the human TPIP-C2 gene derived from TPIP-C2 genomic locus spanning ∼15.3 Kb. Restriction enzymes (RE) used for the study are: E-EcoR I, H-Hind III, B-BamH I. The expected sizes of respective restriction fragments to be detected by the TPIP-C2-ORF probe are calculated from the genomic sequence and are given in the table from left to right as per the map. (C) Testis-specific expression of TPIP-C2 mRNA: Multiple tissue northern (MTN) blot of poly(A^+^) mRNAs from the human tissues was hybridized with [^32^P] labeled 0.603 kb TPIP-C2 ORF-DNA probe. A distinct transcript of ∼2.5 kb is observed in the human testis. (D) MTE array of poly(A^+^) mRNAs from the human tissues and human cancer cell lines were hybridized with [^32^P] labeled 0.603 kb TPIP-C2 ORF-DNA probe. The testis showed strongest signal while other tissues showed very weak signal. PBL: Peripheral blood leucocytes.

We checked the presence of TPIP-C2 sequence in the human genomic DNA. Southern blot analysis of genomic DNA from human peripheral blood lymphocytes using BamH I, EcoR I and Hind III digestions followed by hybridization of ^32^P-TPIP-C2-ORF (603 bp) DNA probe identified genomic DNA fragments of expected sizes based on *in silico* analysis of the genomic organization of the locus (Figure 2A, B). As shown in Figure 2 B, the expected sizes of fragments, which can be detected by TPIP-C2-ORF probe were calculated from the 119.33 kb human genomic sequences on chromosome 13 (NT_024524.13). A search of the cDNA database revealed homology of TPIP-C2 with TPTE2 and TPTE cDNAs (Table S1). TPTE2 and TPIP-C2 cDNAs contain exactly identical coding region, but differ significantly with respect to their 5′-UTR and 3′-UTR regions, thus suggesting possible differential regulation at RNA level with respect to expression and translation of these transcripts in cells.

### Expression of TPIP-C2 mRNA in human tissues

Expression of TPIP-C2 mRNA was studied by using a human multiple tissue northern (MTN) blot and ^32^P-TPIP-C2-ORF DNA probe. A poly(A)^+^ transcript of 2.5 kb was specifically detected in the human testis RNA but not in the spleen, thymus, prostate, ovary, small intestine, colon and peripheral blood leukocytes RNA (Figure 2C). A similar experiment was carried out using a human multiple tissue expression (MTE) array. A prominent signal was again specifically detected in the human testis (Figure 2D, row F, column 8), while RNA from other tissues (Figure 2) was either negative or showed very low levels of expression. This is in agreement with the northern blot result. A number of human cancer cell lines and tissues were negative for TPIP-C2 mRNA expression (Figure 2D, Figure S2A). The full-length ^32^P-TPIP-C2 cDNA probe detected strong signals in almost all tissues because of the SINE and LINE sequences present in the full-length TPIP-C2 cDNA and expression of RNAs containing the SINE and possibly LINE RNAs in the human cells and tissues, this served as an internal control (Figure S2B). These results indicate that TPIP-C2 mRNA is predominantly expressed in human testis.

### Expression of TPIP-C2 mRNA in mouse tissues

**Figure 3:**
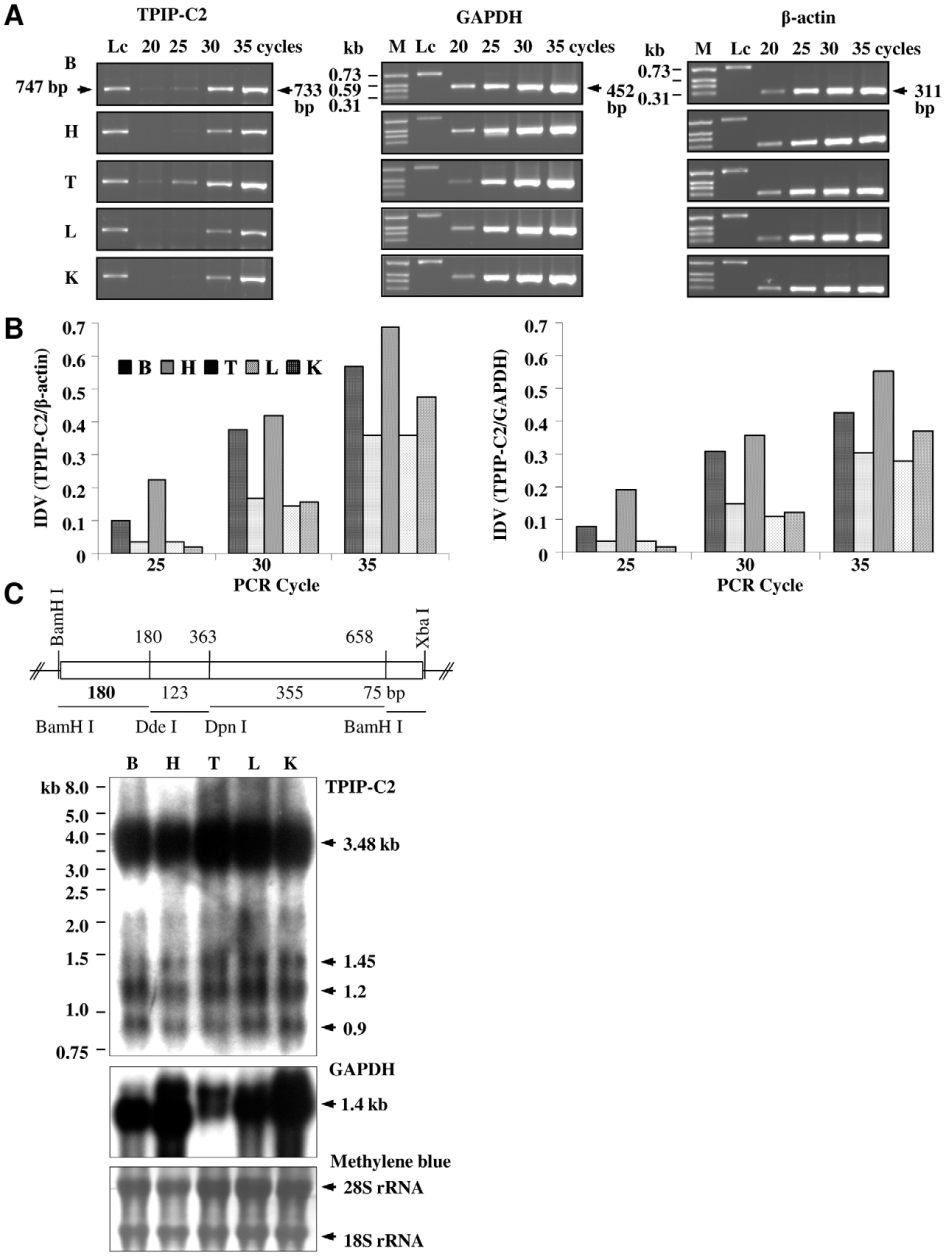
Expression of TPIP-C2 mRNA in mouse tissues. (A) RT-PCR of TPIP-C2 from the mouse tissue RNA. RT-PCR of cellular GAPDH and β-actin mRNAs are shown as internal reference. (B) Normalized RT-PCR signals: the TPIP-C2 mRNA signal was normalized to GAPDH (TPIP-C2/GAPDH) and β-actin (TPIP-C2/β-actin) mRNA signals and plotted. The data represents average of two independent experiments. (C) Schematic representation of the mouse TPIP-C2 probe. The 733 bp mouse TPIP-C2 RT-PCR product was purified, cloned and digested by restriction enzymes (as shown) and the 180 bp BamH I/Dde I DNA fragment was used as the probe. Northern blot hybridization shows TPIP-C2 mRNA expression in the mouse tissues by using random-primed [^32^P] labeled 180 bp TPIP-C2-specific DNA probe. ^32^P-labeled random-primed GAPDH (452 bp) probe was used as an internal control. Methylene blue staining of the ribosomal RNAs on the RNA-filter was used as a loading control. M: Marker, Lc: 20 ng of 0.74 kb DNA as loading control, B: brain, H: heart, T: testis, L: liver and K: kidney.

We checked TPIP-C2 mRNA expression in mouse tissues. RT-PCR analysis was carried out to check whether TPIP-C2-like mRNA is expressed in mouse tissues or not, although no TPIP RNA/cDNA has been reported from mouse till date. We developed a specific RT-PCR assay to distinguish TPIP-C2 transcript from RNAs produced from other TPIP-isoforms. Based on the sequence homology, the ORF-primers (P1 and P2) (Figure S1B) are expected to amplify a 603 bp TPIP-ORF from TPIPα, γ, δ and -C2 transcripts. Therefore, TPIP-C2-specific primers (P3 and P4) were designed to specifically amplify a 733 bp product from cellular TPIP-C2 transcript but not from other related transcripts. Figure S3A (left panel) shows that TPIP-ORF is negative for P3+P4 primers (lane 4) but positive for P1+P2 primers (lane 3). Hence, the 733 bp amplicon (lane 6) is specific for TPIP-C2 cDNA/mRNA. Analysis of RNA from the brain, heart, testis, liver and kidney of adult male mice by RT-PCR showed differential expression of TPIP-C2 mRNA in the mouse tissues (Figure S3A, right panel). The testis and brain showed higher expression of TPIP-C2 mRNA than heart, liver and kidney. The RT-PCR was again carried out for 20, 25, 30, 35 cycles and normalized to GAPDH and β-actin signals. This semi-quantitative RT-PCR analysis showed higher levels of TPIP-C2 mRNA expression in brain and testis in comparison to heart, liver and kidney of mice under normal physiological conditions (Figure 3A, B). The RT-PCR products from brain, heart, testis, liver and kidney were purified, cloned and sequenced to confirm their identity. The TPIP-C2 cDNA sequence (GenBank Accession No. FJ969730) from mouse testis is identical to that obtained from human testis, while other tissues showed single nucleotide changes (Figure S3B, C). These single nucleotide/amino acid changes such as A350G = Thr 99 Ala, G288A = Arg 78 His in heart and T612C = Val 186 Ala in kidney may potentially change the TPIP mRNA coded amino acid sequence in the protein, while some other changes such as T241 T/C, G706A, A665G make no change in the amino acid sequence. Expression of TPIP-C2 mRNA was also confirmed by northern blot hybridization in different mouse tissues by using the TPIP-C2-specific 180 bp (BamH I-Dde I) DNA probe generated from the mouse testis-derived 733 bp RT-PCR product (Figure 3C). It detected a prominent mRNA species of ∼3.48 kb and some additional smaller transcripts of ∼1.45, 1.2 and 0.9 kb in the brain, heart, testis, liver and kidney (Figure 3C). All these results taken together suggest that the TPIP-C2 transcript is expressed at different levels in different tissues of mouse under normal physiological conditions.

### Overexpression of TPIP-C2 in HeLa cells caused inhibition of cell growth/proliferation and induction of apoptosis

**Figure 4:**
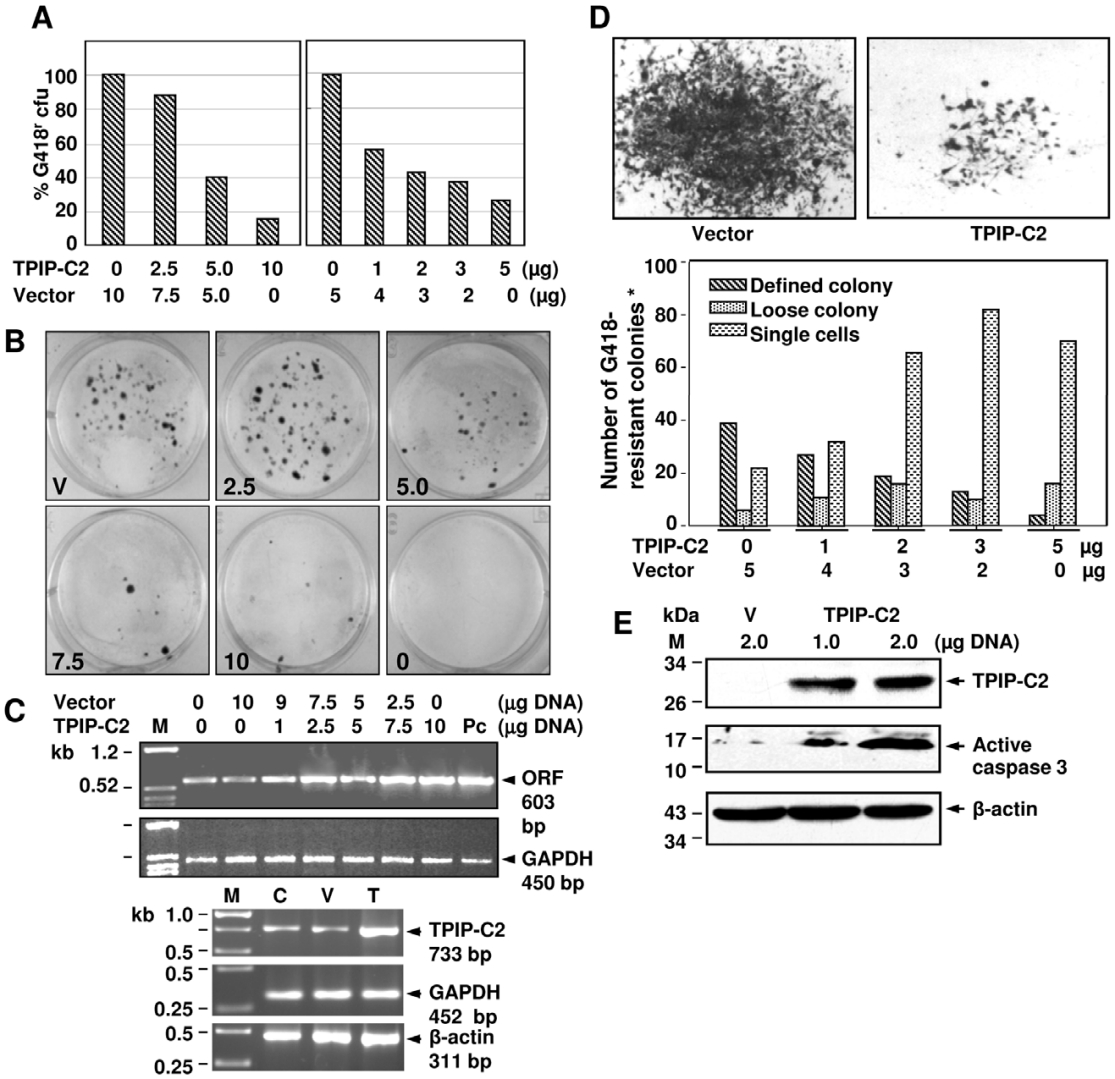
Cell growth/proliferation-suppression by TPIP-C2 cDNA in HeLa cells. (A) HeLa cells were transfected with pCDNA-TPIP-C2 plasmid DNA (2.5, 5.0 and 10 µg DNA in a total of 10 µg of DNA per 6-well plate or with pCDNA-TPIP-C2 plasmid (1, 2, 3 and 5 µg DNA) in a total of 5 µg DNA per well in 6-well plate in duplicates, stable G418-resistant colonies were selected by 0.6 mg/ml G418 for three weeks, and their number was counted. (B) The TPIP-C2-transfected G418-resistant colonies of HeLa cells with respect to transfection of 10 µg of vector DNA (V); 2.5, 5.0, 7.5, 10 µg of TPIP-C2 DNA and negative control (no DNA). (C) Expression of TPIP-C2 mRNA by RT-PCR in the untransfected and transfected HeLa cells. Cells were transfected with the indicated amount of plasmid DNA (total amount of DNA was made 10.0 µg with vector). RT-PCR of cellular GAPDH and β-actin mRNA are shown as internal reference. (D) Morphology of the G418-resistant TPIP-C2 cDNA transfected and vector transfected HeLa cell colonies (upper panel). Quantification of TPIP-C2 transfected G418-resistant HeLa cells showed decrease in the number of defined colonies and increase in the number of single cells with increasing DNA (1, 2, 3 and 5 µg/well) (lower panel). *: Data represent average values of duplicates from two independent experiments. (E) Expression of HA-tagged TPIP-C2 protein in TPIP-C2-HA transfected HeLa cells by Western blot. HeLa cells were transfected with pCDNA-TPIP-C2-HA plasmid (1 and 2 µg) or pCDNA vector (2 µg). Expression of active caspase 3 (cleaved caspase 3) was detected in cell extracts by Western blot analysis. Expression of cellular β-actin protein is shown as an internal reference.

We checked possible cellular function of TPIP-C2 mRNA in human cells. TPIP-C2 cDNA was expressed from pCDNA-TPIP-C2 plasmid in HeLa cells by stable transfection followed by G418-selection resulting into G418-resistant colonies expressing TPIP-C2 mRNA. Overexpression of TPIP-C2 mRNA caused up to 85% decrease in the number of G418-resistant colony formation. This is shown by two independent experiments with two different DNA concentrations (2.5–10 µg DNA per 10 cm plate and 1–5 µg DNA per well of 6 well plate). The suppression of cell growth/proliferation increased from 12% to 85% with increasing amount of cDNA transfected into the cells showing a DNA-dose-dependent effect (Figure 4A, B). The magnitude of cell growth/proliferation suppression (Figure 4A, B) can be correlated with the level of TPIP-C2 mRNA expression measured by RT-PCR in a DNA dose-dependent manner (Figure 4C). Endogenous expression of TPIP-C2 mRNA in HeLa cells is also detected by RT-PCR (Figure 4C lower panel, lane C). The colony morphology and size also significantly changed due to TPIP-C2 mRNA expression and there is a direct correlation between the level of TPIP-C2 mRNA expression and changes in the morphological features of the cells (Figure 4D). TPIP-C2 cDNA transfected colonies are significantly reduced in size with fewer cells. A quantitative representation of these morphological changes in the colonies as a function of increasing amount of TPIP-C2 mRNA expression is shown in Figure 4D (lower panel). The number of defined colonies decreased, the number of single cells increased and the number of loose colonies remained similar suggesting the cell growth/proliferation suppression effects of TPIP-C2 mRNA. The cells appeared to be growth-arrested. The level of endogenous TPIP-C2 mRNA did not alter even after 10 µg vector DNA transfection indicating there was no effect on the cells due to the method of transfection or the vector DNA.

From these results it is clear that TPIP-C2 mRNA is a splice variant (SV) from the human TPIP-locus and the TPIP-C2 protein product is completely identical with the C2-domain of other TPIP isoforms (TPIPα, γ, δ). Therefore, we inserted a HA-tag to make pCDNA-TPIP-C2-HA expression plasmid so that upon transfection, the protein can be detected by western blot using anti-HA antibody. Expression of HA-tagged TPIP-C2 protein is shown in HeLa cells transfected with the HA-tagged plasmid (Figure 4E). Antibody against the HA-tag identified a band of approximately 30 kDa size demonstrating that the TPIP-C2 mRNA was expressed into the TPIP-C2 protein after transfection of the cells. To correlate the cell growth/proliferation suppression effect with over-expression of TPIP-C2 protein, we looked for specific expression of active caspase 3 (17 kDa protein). Caspase 3 is found as an inactive 32 kDa proenzyme and during apoptosis, initiator caspase cleaves the procaspase 3 into 17–19 kDa and ∼12 kDa active forms of caspase 3. Immunoblot analysis of the vector and TPIP-C2-HA transfected cell extracts showed expression of activated caspase 3 in the TPIP-C2-HA transfected cells, which is a hallmark of apoptosis (Figure 4E). The caspase 3 antibody used here is specific for the cleaved 17 kDa protein. Expression of endogenous β-actin protein is shown as internal control. Therefore, the cell growth/proliferation suppression effects can be correlated with expression of the TPIP-C2 protein from the transfected cDNA and TPIP-C2 can cause apoptosis in HeLa cells.

**Figure 5:**
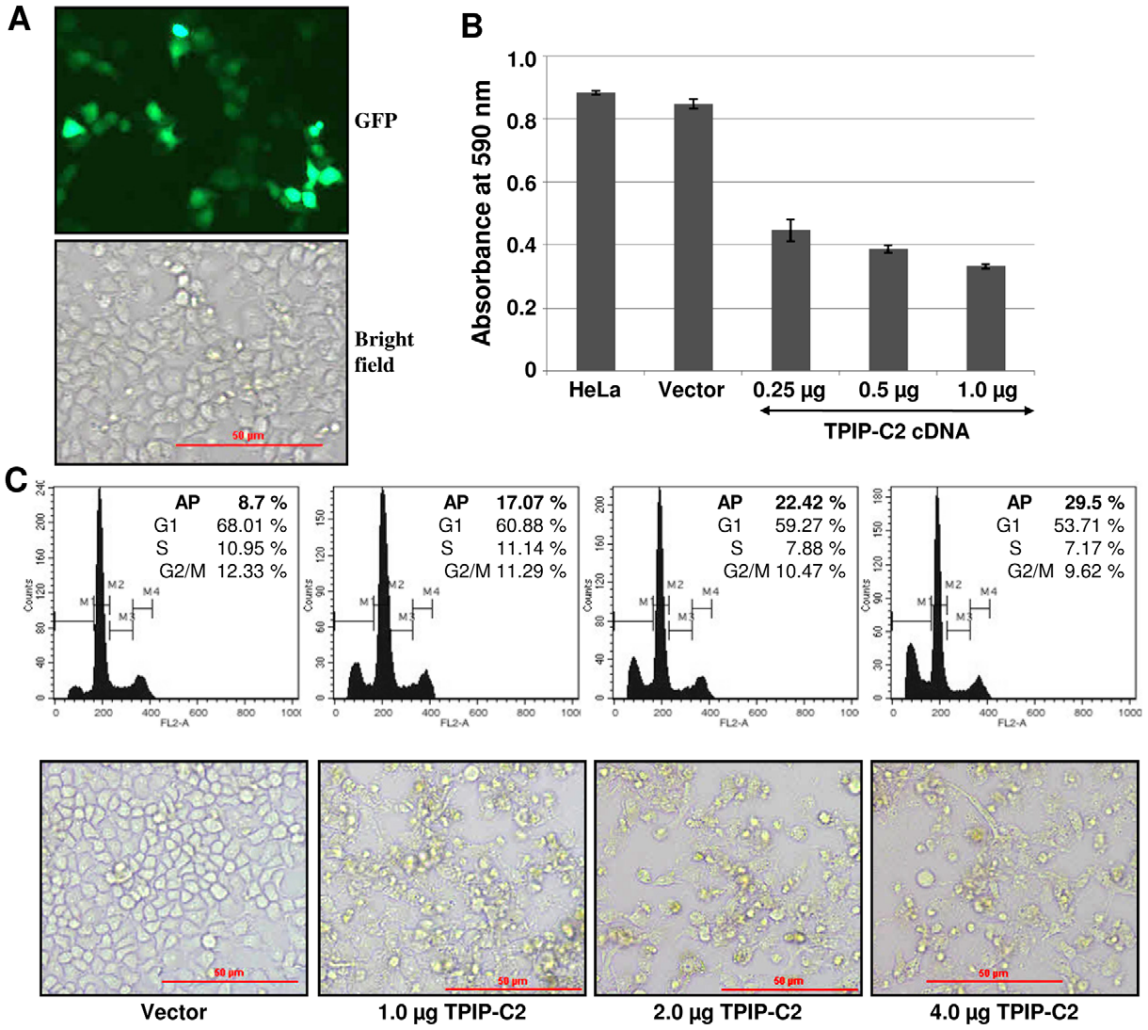
Cell growth/proliferation-suppression and apoptosis by expression of TPIP-C2 in HeLa cells. (A) HeLa cells were transfected with 1 µg CMV-enhanced GFP (pEGFP-N2) plasmid and the transfection efficiency (∼40%–50%) was calculated by counting ∼1000 cells/well. (B) HeLa cells were transfected with pCDNA-TPIP-C2-HA plasmid DNA (0.25, 0.5 and 1.0 µg DNA in a total of 1.0 µg of DNA per 24-well plate in triplicates). Viable cells were quantitated by using MTT assay. Data plotted shows means ± SEM of triplicate wells. (C) Flow cytometric analysis of HeLa cells transfected with TPIP-C2-HA plasmid. DNA was stained with propidium iodide and DNA content was determined by flow cytometry 36 h post-transfection. Data are presented as percentage apoptotic (M1), G1 phase (M2), S phase (M3) and G2/M phase (M4). Morphology of HeLa cells after transfection with TPIP-C2-HA plasmid (1.0, 2.0 and 4.0 µg), showing attached and floating cells visualized under a bright field microscope. This is representative of three independent experiments. AP: Apoptotic, Vector: transfected with 2 µg pCDNA 3.1 plasmid. All experiments were performed 36 h post-transfection.

To further validate the growth/proliferation suppressive effect of TPIP-C2 in HeLa cells, cell proliferation was measured by a cell viability assay (MTT) in TPIP-C2 transfected HeLa cells. The transfection efficiency was ∼40% to 50% as determined by transfection of 1 µg of pEGFP-N2 plasmid in HeLa cells (Figure 5A). As shown in Figure 5B, the cell viability/proliferation inhibition increased from 45% to 62% with the increase in amount of TPIP-C2-HA DNA transfected (0.25 to 1.0 µg) into the cells. As described above, over-expression of TPIP-C2-HA protein caused activation of caspase 3 protein, which is actively involved in apoptosis. The cell cycle status was also analyzed by flow cytometry by measuring the fluorescence from the cells stained with propidium iodide. With the increase in TPIP-C2-HA DNA transfection, the percentage of cells in the G1-phase decreased and the percentage of apoptotic cells in the sub-G1 region increased from 8.7% to 29.5% (Figure 5C). The morphology of the cells transfected with increasing amount of TPIP-C2-HA DNA was observed by bright field microscopy. These cells showed altered morphology in comparison to the vector DNA-transfected cells. The number of floating cells also increased with the increase in amount of transfected DNA (Figure 5, lower panel). These results clearly demonstrate that TPIP-C2 is a potent activator of cell cycle arrest and apoptosis in HeLa cells.

## Discussion

### TPIP-C2 mRNA and its expression in human and mouse tissues

In this study, we report a new isoform of TPIP mRNA, which was isolated from the human testis cDNA library and we named it as TPIP-C2 mRNA, this is in addition to the previously described TPIPα, β, γ and δ isoforms. Mammalian genome maintains the genomic DNA as the blue print of the organism with high degree of consistency and transcribes it into large numbers of RNAs with high degree of fidelity and variability. Primary transcripts from many human genes are processed by different posttranscriptional mechanisms to produce isoforms of RNAs and proteins for carrying out various cellular functions [28]. One of such examples is the human TPIP locus, which is capable of generating multiple mRNA isoforms by alternative splicing. Since there is only a single copy of TPIP gene in the human genome, TPIP-C2 mRNA must be generated by alternative splicing of the TPIP pre-mRNA utilizing cryptic or alternative splice sites. TPIP-C2 cDNA has retrotransposon-derived repeat sequences in the 5′-end (1–324 nt) region, which has no homology with the TPIP and TPTE isoforms but it shows very high sequence similarity with the 2727 nt of TPIP-pseudogene present on chromosome 13. There is also a processed TPIP pseudogene on chromosome 13 (ref|NR_002815.1|). The exon 16 of TPIP-C2 transcript is extended into intron 15 and it also includes a part of the intron 15 in the mature transcript. Thus, it represents an “exon-extended” model of alternative splicing. The extended 5′-exon 16 into the intron 15 forms the 5′-UTR and genomic Southern hybridization with TPIP-ORF probe detected corresponding chromosomal fragments suggesting that TPIP-C2 transcript has been generated by alternative splicing (Figure 1, 2). Alterations in splicing and differential expression of splice-variants (SVs) have been reported for many other genes involved in various cancers and also in other diseases. Novel SVs of PTEN retaining part of introns also have been reported. The PTEN-SVs showed differential expression in heritable and sporadic breast cancers, CS patients and normal healthy controls. Some PTEN-SVs showed higher expression in human fetal tissues in comparison to adult tissues. This is one example showing functional regulation and inactivation of the full-length gene in the inherited cancer syndromes by its splice variants [29].

The TPIP-C2 SINE is also present in 3′-UTR of many other human transcripts indicating its functional significance. Since Alu sequences are abundant and polymorphic in the human genome and most of the human genome is transcriptionally active, we expect presence of Alu sequences in several RNAs. This may have functional consequences and evolutionary significance. SINE and LINE elements located in promoters of some human genes have been reported to contain high-affinity binding sites for hormone receptors, their presence in enhancers suggest role of these mobile DNA elements in regulation of gene expression [30], [31]. The functional significance of LINE and SINE sequences in the 5′-UTR of TPIP-C2 mRNA needs to be further investigated.

TPIP-C2 mRNA is strongly expressed in human testis as a 2.5 kb mRNA and weakly expressed in other tissues (Figure 2). It is differentially expressed in major mouse tissues; the testis shows maximum expression followed by the brain, kidney, heart and liver (Figure 3). TPIP-C2 mRNA expression in mouse tissues differs from expression of other TPTE family members, where the expressed RNA is restricted to testis. The mouse TPIP-C2 mRNA is detected as a 3.5 kb RNA and is distinctly different from PTEN2, a testis-specific RNA of 2.7 kb [32] and other TPTE-isoforms of mouse. As there is no TPIP mRNA reported from mouse yet, this is the first study to identify a TPIP mRNA expressed in a wide variety of mouse tissues under normal physiological conditions. The nucleotide sequence of TPIP-C2 cDNAs generated by RT-PCR from mouse and human testis are highly homologous indicating their evolutionary conservation. However, the relationship of the 1 kb human TPIP-C2 cDNA, the 2.5 kb human mRNA and the 3.5 kb mouse mRNA need further investigation. It will be interesting to study the localization and function of TPIP-C2 isoforms in human cells and mouse tissues. Differences in the size and expression pattern of TPIP-C2 mRNAs in the mouse and human tissues may indicate that they may have different functions in the two species. However, it may be possible that TPIP-C2 mRNA has a generalized function required for normal cellular physiology thus expressed in many cell types/tissues of mouse in contrast to PTEN2, which is a Golgi-associated lipid phosphatase and may have a specialized function during development and differentiation of sperms in testis [32].

### TPIP-C2 protein and its effect

The TPIP-C2 protein belongs to the PTEN-C2 superfamily of phosphatases. The three-dimensional structure of PTEN provides a deep insight into potential mechanisms by which the PTEN phosphatase can recognize and dephosphorylate its 3-phosphate-containing phospholipid substrates. The PTEN-C2 domain lacks the canonical Ca^2+^ ligands, and thus it is similar to the C2 domains of the Ca^2+^-independent protein kinase C (PKC)-isotypes [11]. Classically, C2 domains have a stable β-sheet scaffold, which allows them to fold autonomously. This scaffold allows the emergence of variable loops at the top and bottom of the domain. The β-scaffold probably allows them to bind phospholipids in a Ca^2+^-dependent manner as shown for C2A-domain of synaptotagmin I [33]. PTEN is known to bind phospholipid membranes *in vitro* via its C2-domain and mutation of basic residues in this region reduces PTEN's membrane affinity and its ability to suppress growth of glioblastoma tumor cells [11]. The CBR3 loop in PTEN-C2 domain plays central role in membrane interaction, binding and function of PTEN [34]. TPIP-C2 has a partial phosphatase domain and a C2-domain. It has two conserved hydrophobic residues, Lys-140 and Tyrosine-141 at the CBR3 tip and hydrophobic residues are known to be crucial for insertion in lipid bilayer, therefore, probably it can act in a similar fashion like the PTEN-C2 domain. PTEN causes negative regulation of cell growth/proliferation by downregulating the effects of PI-3kinase. Recent studies have highlighted PTEN as an inducer of apoptosis in cancer cells [35] and the p73/PTEN protein complex can act as a co-activator of apoptosis [36].

Interestingly, over-expression of TPIP-C2 mRNA in HeLa cells also showed similar effects like cell cycle-arrest, suppression of cell growth/proliferation and induction of apoptosis like PTEN (Figure 4 and 5). The effect of TPIP-C2 was observed in a caspase-3 dependent manner as shown by the expression of activated caspase 3 protein (Figure 4). The TPIP-C2 cDNA transfected G418-resistant cells showed more loose colonies and hardly any well defined colony. The morphology of the transfected cells at higher magnification looked more like growth-arrested cells and after transfection, there was hardly any cell proliferation indicating TPIP-C2 mRNA is a potent inhibitor of cell growth and proliferation. Over-expression of TPIP-C2 by transient transfection caused increase in the number of floating cells with severe altered morphology and increased sub-G1 apoptotic population of cells in a dose-dependent manner indicating that TPIP-C2 is a potential inducer of apoptosis (Figure 4 and 5). It was also observed that HeLa cells have endogenous TPIP-C2 mRNA as detected by RT-PCR. This result is in correlation with the MTE blot, where testis showed highest level of expression but other cell types expressed very low levels of TPIP-C2 mRNA (Figure 2).

It has been reported that isolated PTEN C2-domain can inhibit cell migration in U373 cells [18] and during epithelial-to-mesenchymal transition (EMT) of developing chick embryonic mesoderm [37]. It has been also reported that the double phosphatase-deficient PTEN (C124S) [38] and the lipid-phosphatase-dead PTEN (G129E) [39] also inhibited cell invasion in case of glioma cells and bladder cancer cells similar to the wild type PTEN. Recently, it has been reported that PTEN-C2 domain can decrease angiogenesis and VGEF-expression suggesting phosphatase-dependent and -independent functions of PTEN in HepG2 cells [40]. Interestingly, more than 40% of mutations in PTEN have been mapped to the C2-domain [14]. Recently, the PTEN-C2 domain has been reported to cause inhibition of transcription of U6 snRNA [41]. Similarly, the C2 domain of perforin has been shown to mediate Ca^2+^-dependent membrane binding [42]. Natural killer cells and cytotoxic T lymphocytes produce perforin and granzyme. Perforin binds to cell membrane, oligomerizes, makes pores in the membrane and delivers the proapoptotic granzyme to kill virus-infected and neoplastic cells. This example links C2 domain to cell growth inhibition, cytotoxicity and apoptosis. All these observations suggest that C2-domain may be crucial for regulation of cell migration, cell invasion and cell death. The present study shows function of TPIP-C2-domain in negative regulation of cell growth/proliferation and as a potential inducer of apoptosis. TPIP-C2 mRNA/protein may negatively regulate cell growth/proliferation by cell cycle arrest and apoptosis in mammalian cells. Endogenous expression of the TPIP-C2 protein needs to be studied in mammalian cells and tissues for its function. Taken together, TPIP-C2 may be a Ca^2+^-independent PTEN-like C2-domain protein, probably membrane localized, and it induces apoptosis and negatively regulates cell growth/proliferation possibly as a dominant negative molecule by interfering with proteins involved in the interactions of extracellular matrix (ECM) and cell surface associated as well as intracellular proteins. The detailed molecular mechanism of the cell growth/proliferation inhibition by TPIP-C2 mRNA/protein needs further investigation.

## Materials and Methods

### Sequence analysis, Database searches

The 1.019 kb TPIP-C2 cDNA was sequenced and analyzed online using BLAST, ORF finder and RepeatMasker for genomic localization, possible ORF and repeats. CLUSTAL-W amino acid sequence alignment and Kyte and Dolittle hydropathy score were carried out, genomic organization of TPIP-C2 was found by comparing with the 119.33 kb human genomic sequence on chromosome 13 (NT_024524.13) by BioEdit online. The 5′ splice site was identified by comparing TPIP-C2 cDNA with the genomic contig (NT_024524.13). Prediction and comparison of domains was carried out by ELM, SMART through ExPASy Proteomics tools.

### Reagents, cDNA library and antibodies

Molecular biology and tissue culture grade materials and reagents were purchased from Sigma-Aldrich (U S A), New England Biolabs and Promega (U S A), synthetic oligonucleotides were from Life Tech. (U S A) and Microsynth (Switzerland), Lipofectamine™ 2000 (Invitrogen), the human testis λgt11 cDNA library, the human RNA-blots for expression analysis and pEGFP-N2 plasmid were from Clontech (U S A). The radioisotope, [α-^32^P]dATP (specific activity = 4000 Ci/mmole) was from BRIT, India. Anti-β-actin monoclonal antibody (A5316), anti-HA-antibody (H9658), anti-caspase 3 active (C8487), MTT (M2128), Propidium iodide (P4170) were from Sigma-Aldrich.

### DNA constructs, oligonucleotides, genomic DNA, cells and mice

The 1.019 kb TPIP-C2 cDNA was isolated from a λgt11 human testis cDNA library [27] by a 227 bp rat genomic simple repeat DNA probe isolated in this laboratory [43]. The λ-clone was digested by EcoR I and subcloned into EcoR I site of pBluescript vector (pBS-TPIP-C2 plasmid). The TPIP-C2 cDNA was also subcloned into EcoR I site of pCDNA3.1 vector (pCDNA-TPIP-C2 plasmid) for expression in mammalian cells. To check the expression of TPIP-C2 protein, nine aa HA-tag was introduced in the pCDNA-TPIP-C2 plasmid just before the stop codon in the TPIP-C2 ORF by circular PCR using Pfu Turbo DNA polymerase (Stratagene) and Dpn I digestion to make pCDNA-TPIP-C2-HA tagged plasmid. The PCR reaction contained 1× PCR buffer, 25 pmole primers (TPIP-C2-HA primers), 1.0 ng pCDNA-TPIP-C2 template, and 2.5 U Pfu DNA polymerase. The PCR cycle parameters were: initial denaturation at 95°C for 4 min followed by 35 cycles of denaturation at 95°C for 45 sec, annealing at 54°C 45 sec, polymerization at 68°C for 15 min and a final extension of 10 min at 72°C. The 733 bp TPIP-C2 RT-PCR products (described below) from the mouse tissues were cloned into pTZR/T TA-vector (pTZ-TPIP-C2 plasmids). The pBS-TPIP-C2 and pTZ-TPIP-C2 plasmids were used for DNA sequencing. The following forward (f) and reverse (r) oligonucleotides were used as primers for PCR and RT-PCR.

GAPDH: 5′-ACCACAGTCCATGCCATCAC-3′ (f) and 5′-TCCACCACCCTGTTGCTGTA-3′ (r); β-actin: 5′-TTCTACAATGAGCTGCGTGT-3′ (f) and 5′-AGGATCTTCATGAGGTAGTC-3′ (r); TPIP-ORF: 5′-AAGAATAAGCTTATGGTTTGTGCCCTCCTTATTG-3′ (P1), 5′-TCATTGAAGCTTCATTTCTCGCCAAAAAGTATCTCCA-3′ (P2); TPIP-C2: 5′-ATACCATGTATGTTCTTGAACT-3′ (P3) and 5′- GGATTGGAGAGCGGGGATT-3′ (P4). TPIP-C2-HA: 5′-TACCCATACGATGTTCCAGATTACGCTTGACTTCCAATGATGTTGTAG-3′ (f) and 5′-TCAAGCGTAATCTGGAACATCGTATGGGTATTTCTCGCCAAAAAGTATCTC-3′ (r).

Human genomic DNA was purified from human peripheral blood lymphocytes, human cervical carcinoma (HeLa) cells were from ATCC (U S A) and 12–14 weeks old Swiss albino male mice were obtained from the Animal house of Jawaharlal Nehru University. All experimental protocols involving the use of animals were reviewed by the Institutional Animal Ethics Committee (IAEC); registration No. 19/1999 (CPCSEA), dated 10.03.1999, IAEC code No 14/2006, Jawaharlal Nehru University, New Delhi, India. The molecular biology methods were followed as described earlier [44].

### Northern blot hybridization

Total cellular RNA was isolated from fresh mouse tissues with a few modifications. Northern blot hybridization was carried out as described earlier [43]. Briefly, 20 µg of total RNA was denatured and resolved in 1.5% formaldehyde-agarose gel, transferred to nylon membrane (Biodyne A, Pall) by vacuum-blotting and the RNA-filter was pre-hybridized and hybridized in 50% formamide-based hybridization mixture with random-primed ^32^P-labeled mouse TPIP-C2-specific 180 bp cDNA probe (specific activity >10^8^ cpm/µg) for 16–18 h at 42°C. The RNA-filter was stringently washed and exposed to X-ray film with intensifying screens at −80°C for 2–3 days and developed for autoradiography. The human multiple tissue northern (MTN, Clontech, 7759-1) and multiple tissue expression (MTE, Clontech, 7775-1) array RNA blots (Clonetech, U S A) were hybridized with ^32^P-labeled TPIP-C2-ORF DNA probe as per the manufacturer's instructions. The amount of RNA per lane on Poly A^+^ MTN blot used in Figure 2B is adjusted to obtain consistent signal for a house keeping gene across all lanes. For this reason, the actual amount of RNA loaded may vary slightly between samples.

### Southern blot hybridization

Genomic DNA was isolated from human peripheral blood from healthy individual with consent. Twenty microgram genomic DNA was digested by BamH I, EcoR I and Hind III, electrophoresed by agarose gel and southern hybridized with random primed ^32^P-TPIP-C2-ORF DNA probe and detected by autoradiography [44].

### Transfection of HeLa cells

HeLa cells were seeded at 0.15×10^6^ cells/ml density in 2 ml in 6-well plates, 18 h before transfection and transfected by calcium phosphate or Lipofectamine™ 2000 in duplicates with pCDNA-TPIP-C2 plasmid DNA (i.e., 2.5, 5.0 and 10 µg) in a total amount of 10 µg DNA per 10 cm plate or 0, 1, 2, 3 and 5 µg DNA in a total amount of 5 µg DNA per well in six-well plate for 12 h by calcium phosphate method. The pCDNA3.1 (+) vector DNA was used to compensate the total amount of DNA up to equal amount per well. The transfection medium was replaced by fresh medium and cells were allowed to grow for 24 h followed by addition of selection medium containing 0.6 mg/ml geneticin (G418, Life Tech., U S A) for 3 weeks. The medium was changed every third day until G418-resistant colonies developed, which were then fixed by methanol, stained with Giemsa stain, observed, counted and photographed by a phase-contrast microscope. HeLa cells were transiently transfected in duplicates with different amounts (1 to 10 µg) of pCDNA-TPIP-C2 plasmid DNA in a total amount of 10 µg of DNA compensated with the vector DNA by calcium phosphate method and 24 h post-transfection, cells were harvested, total cellular RNA was isolated and used for RT-PCR. HeLa cells were also transiently transfected with pCDNA-TPIP-C2-HA plasmid DNA, pEGFP-N2 or pCDNA 3.1 (vector) with 2 µg and 4 µg DNA per well in six-well plate for 6 h in serum free medium by using Lipofectamine™ 2000 reagent as per the supplier's instructions. After 36 h post-transfection the cells were visualized and photographed in bright field and fluorescence settings of the microscope. Cells were harvested and processed for western blot analysis or cell cycle analysis by FACS.

### Western blot analysis

The HeLa cells were harvested 36 h after transfection, washed with cold PBS and extracted in lysis buffer (20 mM Tris-HCl, pH 8, 250 mM NaCl, 1 mM DTT, 2 mM EDTA, 0.5% NP-40, 1% Triton X-100, 10 µg/ml leupeptin, 10 µg/ml aprotinin, 0.5 mg/ml benzamidine, 100 mM PMSF and 2 mM sodium orthovanadate). After 30-min incubation on ice, the lysates were cleared by centrifugation at 12000 rpm for 30 min at 4° C. Protein concentration was estimated using Bradford assay. Approximately, 30–60 µg protein was resolved by 12% SDS-PAGE, transferred to nitrocellulose membrane (Bio-Rad). Western blots were carried out by using anti-HA antibody for TPIP-C2-HA protein, anti-caspase 3 for active caspase 3 and anti-β-actin antibody. The blots were developed by Super Signal West Pico Chemiluminescence reagent (Pierce).

### RNA isolation and RT-PCR

RNA was isolated from mouse tissues and human cells. First strand cDNAs were synthesized from 1.0 µg of total RNA with 0.5 µg of oligo dT [5′-(dT)_15_-3′] primer, 0.5 mM dNTPs, 20 U RNasin and 100 U of M-MLV Reverse Transriptase in 1× M-MLV RT-reaction buffer in 25 µl. First strand cDNA mixture was used as template and pCDNA-TPIP-C2 plasmid was used as positive control (Pc) for PCR. The reaction contained 1× PCR buffer (75 mM Tris.Cl pH 9.0, 50 mM KCl, 20 mM (NH_4_)_2_SO_4_, 0.2 mM dNTPs, 2.0 mM MgCl_2_, 25 pmole primers (12.5 pmole each), 1.0–5.0 µl 1^st^ strand cDNA templates and 1.0 U Taq DNA polymerase. The PCR cycle parameters were initial denaturation at 94°C for 4 min followed by 20–35 cycles of denaturation at 94°C for 45 sec, annealing at 68°C (TPIP-ORF) or 51°C (TPIP-C2 and β-actin), 60°C (GAPDH) for 45 sec-1 min, polymerization at 72°C for 1 min and a final extension of 10 min at 72°C. TPIP-ORF primers amplified a 603 bp C2-ORF from human TPIPα, γ and δ, TPTEα, β, γ mRNAs, whereas, TPIP-C2 primers are specific for the TPIP-C2 transcript and amplified a 733 bp amplicon. The 311 bp β-actin and 452 bp GAPDH products were used as control and normalization signals. The PCR products (1/10^th^ for GAPDH, β-actin and 1/5^th^ for TPIP-C2) were electrophoresed in 1.5% agarose-TBE gels, photographed by an AlphaImager 3400 gel-documentation system and densitometry of specific DNA bands was carried out (Integrated Density Value or IDV) by the AlphaImager software.

### Cell proliferation measurement by cell viability assay (MTT)

Seventy thousand cells were plated per well in 24 well plate and transfected in triplicates with 0.25 µg, 0.5 µg and 1.0 µg of TPIP-C2-HA plasmid for 6 h by using Lipofectamine™ 2000 reagent in serum free medium as per the supplier's instructions. After 36 h post-transfection 100 µl MTT (5 mg/ml) was added to each well and incubated at 37°C. After 2 h MTT was solubilized by addition 500 µl of MTT solvent (5 mM HCl, 0.1% Nonidet P-40 in isopropanol) and incubated for another 1 h at 37°C. The absorbance was measured spectrophotometrically at 590 nm and plotted. The data were represented as mean ± SEM from three independent transfection experiments and each experiment was repeated twice.

### Cell cycle analysis

HeLa cells were transfected with TPIP-C2-HA plasmid in six well plates as described above and harvested 36 h post-transfection. Cells were trypsinized, washed twice with ice-cold PBS, fixed in 70% ethanol in ice-cold PBS for 2 h at 4°C. For flow cytometric analysis, the cells were incubated with 0.1 mg/ml RNase A at 37°C for 1 h, stained with 50 µg/ml propidium iodide for 15–20 min on ice, and then measured by flow cytometry using FACSCalibur (Becton Dickinson, San Jose, CA, U S A) Cell Quest software for acquisition and analysis. A minimum of 20,000 events were recorded for each sample.

## Supporting Information

Figure S1 (A) TPIP-C2 cDNA (1.019 kb inserted DNA) is cloned at EcoR I site of pBSKII^+^ (pBluescript) vector. (B) TPIP-C2 cDNA nucleotide sequence and predicted amino acid sequence. (C) TPIP-C2-SINE and TPIP-C2-LINE sequences are compared with AluSx and LINE (L3/CR1) repeat sequences, respectively. The transition and transversion in the sequences are indicated. (D) Comparison of TPIP-C2 with the TPIP genomic contig on human chromosome 13. TPIP exons and introns with respect to human DNA sequences from the clone RP11-408K19 on chromosome 13 [AL590076]. The introns are depicted as dotted line and exons as black boxes. The number below the exons represents length of respective exon. The exon sequences corresponding to the chromosome 13 genomic regions are mentioned (upper panel). TPIP-C2 exons and introns are with respect to human DNA sequence from the clone RP11-408K19 on chromosome 13 [AL590076] (lower panel).
(PDF)

Figure S2 (A) Sources of human cells and tissues for the mRNAs used in the dot blots. (B) Hybridization of the MTE-blot with [^32^P] labeled 1.019 kb full length TPIP-C2 cDNA probe showing homologous RNA expressions due to presence of SINE/LINE sequences present in the full length TPIP-C2 cDNA. Almost all samples show the signals. A number of controls are indicated at the right hand side of the MTE blot.
(PDF)

Figure S3 Expression of TPIP-C2 mRNA in mouse tissues. (A) TPIP-ORF amplicon is 605 bp (339–921 nt) and TPIP-C2 amplicon is 733 bp (285–2017 nt) as shown by PCR/RT-PCR products amplified by using P1+P2 and P3+P4 primers, respectively. TPIP-ORF primers contain extra flanking sequences and Hind III site at their 5′-ends, thus, instead of 582 bp amplicon, it amplifies 605 bp DNA. Establishment of TPIP-C2-specific PCR assay (top panel), Lane 1 and 2: pCDNA-TPIP-C2 plasmid control for TPIP-ORF and TPIP-C2 PCR, lane 3 and 5: TPIP-ORF PCR with ORF 339–921 and ORF 285–1087 templates, lane 4: ORF 339–921 and β-actin templates were mixed and PCR-amplified for TPIP-C2 and β-actin (311 bp) products with respective primers, lane 6: TPIP-C2 PCR from ORF 285–1087 template, TPIP-C2 plasmid PCR with 10^4^–10^1^ copies of the plasmid template (bottom panel) showing the sensitivity of PCR reaction at 10^1^ copy number. (B) Chromatograms of DNA sequencing of one each type of representative RT-PCR products cloned from the mouse tissues showing single nucleotide changes from the expected sequence. (C) The amino acid changes with respect to the single nucleotide changes in TPIP-C2 193 aa. ORF are shown. M: Marker, Pc: positive control, Lc: 20 ng of 0.74 kb DNA as a loading control for densitometric measurements, B: brain, H: Heart, T: testis, L: liver and K: kidney of mouse.
(PDF)

Table S1 Homology of TPIP-C2 CDNA with human genome.
(DOC)

Table S2 Presence of TPIP-C2 SINE and LINE in human transcripts.
(DOC)

## References

[1] Steck PA, Pershouse MA, Jasser SA, Yung WK, Lin H (1997). Identification of a candidate tumor suppressor gene, MMAC1, at chromosome 10q23.3 that is mutated in multiple advanced cancers.. Nat Genet.

[2] Li DM, Sun H (1997). TEP1, encoded by a candidate tumor suppressor locus, is a novel protein tyrosine phosphatase regulated by transforming growth factor 1.. Cancer Res.

[3] Li J, Yen C, Liaw D, Podsypanina K, Bose S (1997). PTEN, a putative protein tyrosine phosphatase gene mutated in human brain, breast, and prostate cancer.. Science.

[4] Liaw D, Marsh DJ, Li J, Dahia PL, Wang SI (1997). Germline mutations of the PTEN gene in Cowden disease, an inherited breast and thyroid cancer syndrome.. Nat Genet.

[5] Gustafson S, Zbuk KM, Scacheri C, Eng C (2007). Cowden syndrome.. Semin Oncol.

[6] Sansal I, Sellers WR (2004). The biology and clinical relevance of the PTEN tumor suppressor pathway.. J Clin Oncol.

[7] Wang X, Jiang X (2008). PTEN: a default gate-keeping tumor suppressor with a versatile tail.. Cell Res.

[8] Carnero A (2010). The PKB/AKT pathway in cancer.. Curr Pharm Des.

[9] Myers MP, Stolarov JP, Eng C, Li J, Wang SI (1997). PTEN, the tumor suppressor from human chromosome 10q23, is a dual-specificity phosphatase.. Proc Natl Acad Sci U S A.

[10] Maehama T, Dixon JE (1998). The tumor suppressor, PTEN/MMAC1, dephosphorylates the lipid second messenger, phosphatidylinositol 3,4,5-triphosphate.. J Biol Chem.

[11] Lee JO, Yang H, Georgescu MM, Di Cristofano A, Maehama T (1999). Crystal structure of the PTEN tumor suppressor: implications for its phosphoinositide phosphatase activity and membrane association.. Cell.

[12] Georgescu MM, Kirsch KH, Kaloudis P, Yang H, Pavletich NP (2000). Stabilization and productive positioning roles of the C2 domain of PTEN tumor suppressor.. Cancer Res.

[13] Vazquez F, Ramaswamy S, Nakamura N, Sellers WR (2000). Phosphorylation of the PTEN tail regulates protein stability and function.. Mol Cell Biol.

[14] Eng C (2003). PTEN: One gene, many syndromes.. Hum Mutat.

[15] Das S, Dixon JE, Cho W (2003). Membrane-binding and activation mechanism of PTEN.. Proc Natl Acad Sci U S A.

[16] Leslie NR, Yang X, Downes CP, Weijer CJ (2005). The regulation of cell migration by PTEN.. Biochem Soc Trans.

[17] Odriozola L, Singh G, Hoang T, Chan AM (2007). Regulation of PTEN activity by its carboxyl-terminal autoinhibitory domain.. J Biol Chem.

[18] Raftopoulou M, Etienne-Manneville S, Self A, Nicholls S, Hall A (2004). Regulation of cell migration by the C2 domain of the tumor suppressor PTEN.. Science.

[19] Meuillet EJ, Mahadevan D, Berggren M, Coon A, Garth P (2004). Thioredoxin-1 binds to the C2 domain of PTEN inhibiting PTEN's lipid phosphatase activity and membrane binding: a mechanism for the functional loss of PTEN's tumor suppressor activity.. Arch Biochem Biophys.

[20] Mehenni H, Lin-Marq N, Buchet-Poyau K, Reymond A, Collart MA (2005). LKB1 interacts with and phosphorylates PTEN: a functional link between two proteins involved in cancer predisposing syndromes.. Hum Mol Genet.

[21] Zhou M, Gu L, Findley HW, Jiang R, Woods WG (2003). PTEN reverses MDM2-mediated chemotherapy resistance by interacting with p53 in acute lymphoblastic leukemia cells.. Cancer Res.

[22] Tang Y, Eng C (2006). PTEN autoregulates its expression by stabilization of p53 in a phosphatase-independent manner.. Cancer Res.

[23] Chen H, Rossier C, Morris MA, Scott HS, Gos A (1999). A testis-specific gene, TPTE, encodes a putative transmembrane tyrosine phosphatase and maps to the pericentromeric region of human chromosomes 21 and 13, and to chromosomes 15, 22, and Y.. Hum Genet.

[24] Guipponi M, Yaspo ML, Riesselman L, Chen H, Sario ADe (2000). Genomic structure of a copy of the human TPTE gene which encompasses 87 kb on the short arm of chromosome 21.. Human Genet.

[25] Tapparel C, Reymond A, Girardet C, Guillou L, Lyle R (2003). The TPTE gene family: cellular expression, subcellular localization and alternative splicing.. Gene.

[26] Walker SM, Downes CP, Leslie NR (2001). TPIP: a novel phosphoinositide 3-phosphatase.. Biochem J.

[27] Bajaj GD (2002). Molecular cloning and characterization of human cDNAs by a simple repeat DNA probe: Identification of novel candidate genes..

[28] Lander ES, Linton LM, Birren B, Nusbaum C, Zody MC (2001). Initial sequencing and analysis of the human genome.. Nature.

[29] Agrawal S, Eng C (2006). Differential expression of novel naturally occurring splice variants of PTEN and their functional consequences in Cowden syndrome and sporadic breast cancer.. Hum Mol Genet.

[30] Babich V, Aksenov N, Alexeenko V, Oei SL, Buchlow G (1999). Association of some potential hormone response elements in human genes with Alu family repeats.. Gene.

[31] Yang Z, Boffelli D, Boonmark N, Schwartz K, Lawn R (1998). Apolipoprotein(a) gene enhancer resides within a LINE element.. J Biol Chem.

[32] Wu Y, Dowbenko D, Pisabarro M, Dillard-Telm L, Koeppen H (2001). PTEN 2, a Golgi-associated testis-specific homologue of the PTEN tumor suppressor lipid phosphatase.. J Biol Chem.

[33] Rizo J, Südhof TC (1998). C2 domain, structure and function of a universal Ca^2+^-binding domain.. J Biol Chem.

[34] Rahdar M, Inoue T, Meyer T, Zhang J, Vazquez F (2009). A phosphorylation-dependent intramolecular interaction regulates the membrane association and activity of the tumor suppressor PTEN.. Proc Natl Acad Sci U S A.

[35] Lee JJ, Kim BC, Park MJ, Lee YS, Kim YN (2011). PTEN status switches cell fate between premature senescence and apoptosis in glioma exposed to ionizing radiation.. Cell Death Differ.

[36] Lehman JA, Waning DL, Batuello CN, Cipriano R, Kadakia MP (2011). Induction of apoptotic genes by a p73-PTEN complex in response to genotoxic stress.. J Biol Chem.

[37] Maier D, Jones G, Li X, Schonthal AH, Gratzl O (1999). The PTEN lipid phosphatase domain is not required to inhibit invasion of glioma cells.. Cancer Res.

[38] Gildea JJ, Herlevsen M, Harding MA, Gulding KM, Moskaluk CA (2004). PTEN can inhibit in vitro organotypic and in vivo orthotopic invasion of human bladder cancer cells even in the absence of its lipid phosphatase activity.. Oncogene.

[39] Li AG, Piluso LG, Cai X, Wei G, Sellers WR (2006). Mechanistic insights into maintenance of high p53 acetylation by PTEN.. Mol Cell.

[40] Tian T, Nan KJ, Wang SH, Liang X, Lu CX (2010). PTEN regulates angiogenesis and VEGF expression through phosphatase-dependent and -independent mechanisms in HepG2 cells.. Carcinogenesis.

[41] Cabarcas S, Watabe K, Schramm L (2010). Inhibition of U6 snRNA transcription by PTEN.. Online J Biol Sci.

[42] Law RH, Lukoyanova N, Voskoboinik I, Caradoc-Davies TT, Baran K (2010). the structural basis for membrane binding and pore formation by lymphocyte perforin.. Nature.

[43] Dey I, Rath PC (2005). A novel rat genomic simple repeat DNA with RNA-homology shows triplex (H-DNA)-like structure and tissue-specific RNA expression.. Biochem Biophys Res Commun.

[44] Sambrook J, Russell DW (2001). Molecular Cloning: A Laboratory Manual..

