# Supplementary material for: A Novel Human TPIP Splice-Variant (TPIP-C2) mRNA, Expressed in Human and Mouse Tissues, Strongly Inhibits Cell Growth in HeLa Cells
Source: PLoS One. 2011 Dec 2;6(12):e28433. doi: 10.1371/journal.pone.0028433 (PMC3229583; doi:10.1371/journal.pone.0028433)
Supplement: Figure S1 — (A) TPIP-C2 cDNA (1.019 kb inserted DNA) is cloned at EcoR I site of pBSKII+ (pBluescript) vector. (B) TPIP-C2 cDNA nucleotide sequence and predicted amino acid sequence. (C) TPIP-C2-SINE and TPIP-C2-LINE sequences are compared with AluSx and LINE (L3/CR1) repeat sequences, respectively. The transition and transversion in the sequences are indicated. (D) Comparison of TPIP-C2 with the TPIP genomic contig on human chromosome 13. TPIP exons and introns with respect to human DNA sequences from the clone RP11-408K19 on chromosome 13 [AL590076]. The introns are depicted as dotted line and exons as black boxes. The number below the exons represents length of respective exon. The exon sequences corresponding to the chromosome 13 genomic regions are mentioned (upper panel). TPIP-C2 exons and introns are with respect to human DNA sequence from the clone RP11-408K19 on chromosome 13 [AL590076] (lower panel). (PDF) [file pone.0028433.s001.pdf]

Supplementary Figure 1

A

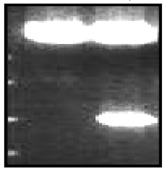

B

```
   1 ggtttttttttactattatccaaaaaaattataaaagacatgaattgttgctttcttat

  61 agatttagtaaaaacctcatcgtgtccttttgtggagagatggtgagagatatttttact
                                            M
 121 gtaaatgtaatttgcgtatttttcacctgtgaggcggaggttgcaatgagccaagattgt
                             145            AluSx    M
 181 gccactgcactccagcctgcgtgacacagcaagaccctgtctcaaaaaaaaagaaagaaa
                                    *
                                                         P3:285-306
 241 atcaagttaaaatgcactccttgaatatttctgagtgtatttttataccatgtatgttct
     241                            268                L3/CR1
                                    *
                                                        P1:339-361
 301 tgaactaacttcttttcttttctaggaagaaccgggactatggtttgtgccctccttatt
     *                            L G328R T G T M V C A L L I
 361 gcctccgaaatatttttaactgccgaggaaagcctatattatttggagaaaggcgaacc
   8 A S E I F L T A E E S L Y Y F G E R R T
 421 aataaaacccacagcaataaatttcagggagtagaaactccttctcagaatagatatgtt
  28 N K T H S N K F Q G V E T P S Q N R Y V
 481 ggatatttgcacaagtgaaacatctctacaactggaatctccctccaagacggatactc
  48 G Y F A Q V K H L Y N W N L P P R R I L
 541 tttataaaaagattcattatttattcgattcgtggtgatgtatgtgatctaaaagtccaa
  68 F I K R F I I Y S I R G D V C D L K V Q
 601 gtagtaatggagaaaaaggttgtcttttccagtacttcattaggaaattgttcgatattg
  88 V V M E K K V V F S S T S L G N C S I L
 661 catgacattgaaacagacaaagtattaattaatgtatatgacggtccacctctgtatgat
 108 H D I E T D K V L I N V Y D G P P L Y D
 721 gatgtgaaagtgcagtttttctcttcgaatcttcctaaatactatgacaattgtccattt
 128 D V K V Q F F S S N L P K Y Y D N C P F
 781 ttcttctggttcaacacgtcttttattcaaaataacaggctttgtctaccaagaaatgaa
 148 F F W F N T S F I Q N N R L C L P R N E   P2:921-896
 841 ttggataatccacataaacaaaaagcatggaaaatttatccaccagaatttgctgtggag
 168 L D N P H K Q K A W K I Y P P E F A V E
                            921                   P4
 901 atacttttggcgagaaatgacttccaatgatgttgtagctggatccgactaagtatagc
 188 I L F G E K *                  P4:999        1017
 961 tccccctttccccttctgggaaagaattatgttctttccaatccccgctctccaatcccg 1019
```

C

Supplementary Figure 1

Comparison of TPIP-C2 SINE with AluSx

```
TPIP-C2 145 ACCTGTGAGGCGGAGGTTGCAATGAGCCAAGATTGTGCCACTGCACTCCA 194
               i v              i      i    i i
AluSx   204 ACCCGGGAGGCGGAGGTTGCAGTGAGCCGAGATCGCGCCACTGCACTCCA 253

TPIP-C2 195 GCCTGCGTGACACAGCAAGACCCTGTCTCAAAAAAAAAGAAAGAAAA 241
                 v i    v  i   i i             i   i
AluSx   254 GCCTGGGCGACAGAGCGAGACTCCGTCTCAAAAAAAAAAAAAAAAAA 300
```

Comparison of TPIP-C2 LINE with L3/CR1

```
TPIP-C2 268 TTTCTGAGTGTATTTTTA----TACCATGTATGTTCTTGAACTAACTTCT 313
                v                   v    i           i  vi
L3/CR1  268 TTTCAGAGTGTATTTTTATTTTTAACATGC---TTCTTGAATTATTTTCT 222

TPIP-C2 314 TT-----TCTTTTCTAGGAA 328
                       i
L3/CR1  221 TTACTCATCTTCTCTAGGAA 202
```

i: transition, v: transversion, -: gap

D

TPIP genomic contig

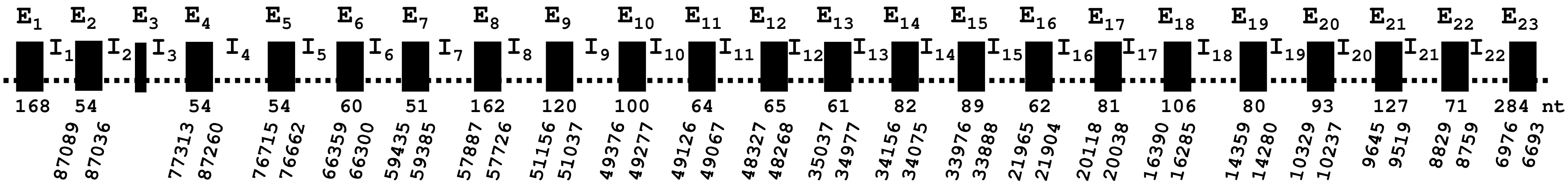

TPIP-C2

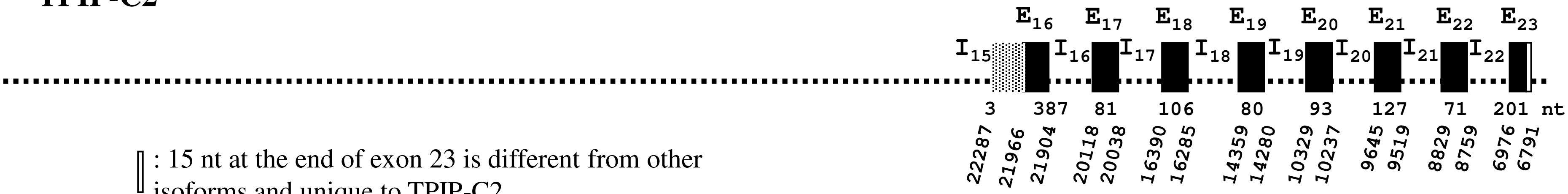

▯ : 15 nt at the end of exon 23 is different from other isoforms and unique to TPIP-C2
