# Supplementary material for: A Novel Human TPIP Splice-Variant (TPIP-C2) mRNA, Expressed in Human and Mouse Tissues, Strongly Inhibits Cell Growth in HeLa Cells
Source: PLoS One. 2011 Dec 2;6(12):e28433. doi: 10.1371/journal.pone.0028433 (PMC3229583; doi:10.1371/journal.pone.0028433)

Supplementary Figure 2

A

Whole brain	Cerebellum left	Substantia nigra	heart	Oesophagus	Colon transverse	kidney	lung	Liver	Leukemia HL-60	Fetal brain	Yeast total RNA
Cerebral cortex	Cerebellum right	Nucleus accumbens	Aorta	Stomah	Colon descending	Skeletal muscle	placenta	pancreas	HeLa S3	Fetal heart	Yeast tRNA
Frontal lobe	Corpus callosum	thalamus	Atrium left	Duodenum	rectum	spleen	bladder	Adrenal gland	Leukemia K-562	Fetal kidney	*E. Coli* rRNA
Parietal lobe	amygdala	Pituitary gland	Atrium right	Jejenum		Thymus	Uterus	Thyroid gland	Leukemia MOLT-4	Fetal liver	*E. Coli* DNA
Occipital lobe	Caudate nucleus	Spinal cord	ventricle left	Ilelum		Periphera l blood leucocyte	Prostte	Salivary gland	Burkitt's lymphoma Raji	Fetal spleen	Poly r(A)
Temporal lobe	Hippocampus		ventricle right	Ileocecum		Lymph node	Testis	Mmary gland	Burkitt's lymphoma Daudi	Fetal thymus	Human Cot-1 DNA
Paracentral gyrus of cerebral cortex	medulla oblongate		Intervent ricular septum	Appendix		Bone marrow	ovary		Colorectal carcinoma SW480	Fetal lung	Human DNA 100 ng
pons	putamen		Apex of the heart	Colon ascending		trachea			Lung carcinoma A549		Human DNA 500 ng

B

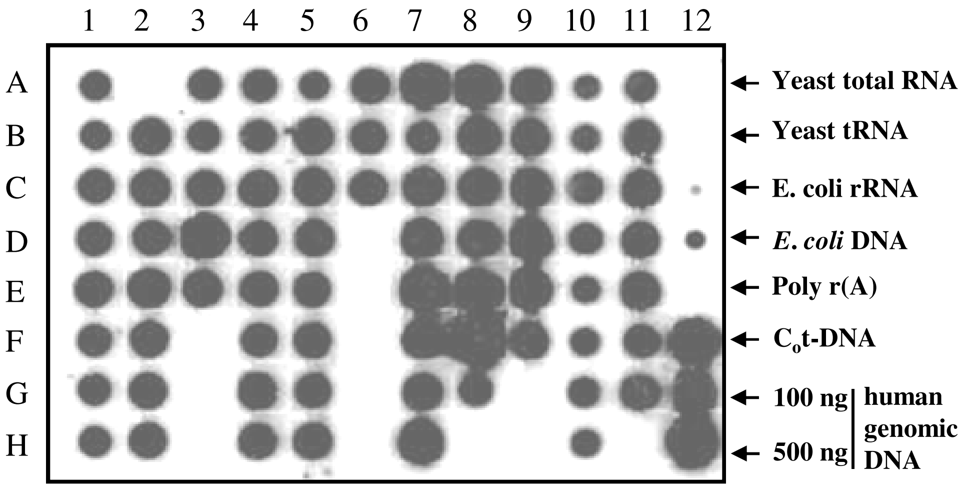

Supplement: Figure S2 — (A) Sources of human cells and tissues for the mRNAs used in the dot blots. (B) Hybridization of the MTE-blot with [32P] labeled 1.019 kb full length TPIP-C2 cDNA probe showing homologous RNA expressions due to presence of SINE/LINE sequences present in the full length TPIP-C2 cDNA. Almost all samples show the signals. A number of controls are indicated at the right hand side of the MTE blot. (PDF) [file pone.0028433.s002.pdf]
