# Supplementary material for: A Novel Human TPIP Splice-Variant (TPIP-C2) mRNA, Expressed in Human and Mouse Tissues, Strongly Inhibits Cell Growth in HeLa Cells
Source: PLoS One. 2011 Dec 2;6(12):e28433. doi: 10.1371/journal.pone.0028433 (PMC3229583; doi:10.1371/journal.pone.0028433)
Supplement: Figure S3 — Expression of TPIP-C2 mRNA in mouse tissues. (A) TPIP-ORF amplicon is 605 bp (339–921 nt) and TPIP-C2 amplicon is 733 bp (285–2017 nt) as shown by PCR/RT-PCR products amplified by using P1+P2 and P3+P4 primers, respectively. TPIP-ORF primers contain extra flanking sequences and Hind III site at their 5′-ends, thus, instead of 582 bp amplicon, it amplifies 605 bp DNA. Establishment of TPIP-C2-specific PCR assay (top panel), Lane 1 and 2: pCDNA-TPIP-C2 plasmid control for TPIP-ORF and TPIP-C2 PCR, lane 3 and 5: TPIP-ORF PCR with ORF 339–921 and ORF 285–1087 templates, lane 4: ORF 339–921 and β-actin templates were mixed and PCR-amplified for TPIP-C2 and β-actin (311 bp) products with respective primers, lane 6: TPIP-C2 PCR from ORF 285–1087 template, TPIP-C2 plasmid PCR with 104–101 copies of the plasmid template (bottom panel) showing the sensitivity of PCR reaction at 101 copy number. (B) Chromatograms of DNA sequencing of one each type of representative RT-PCR products cloned from the mouse tissues showing single nucleotide changes from the expected sequence. (C) The amino acid changes with respect to the single nucleotide changes in TPIP-C2 193 aa. ORF are shown. M: Marker, Pc: positive control, Lc: 20 ng of 0.74 kb DNA as a loading control for densitometric measurements, B: brain, H: Heart, T: testis, L: liver and K: kidney of mouse. (PDF) [file pone.0028433.s003.pdf]

Supplementary Figure 3

A

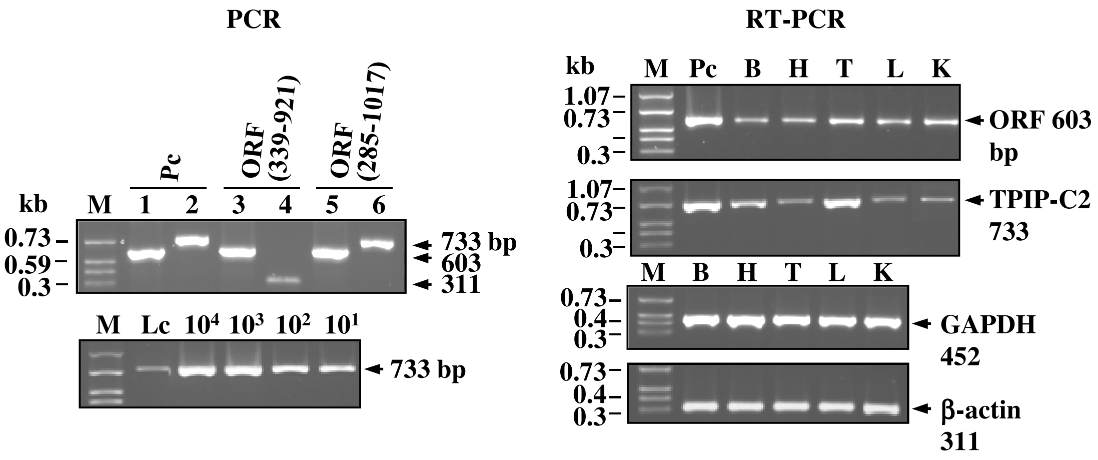

B

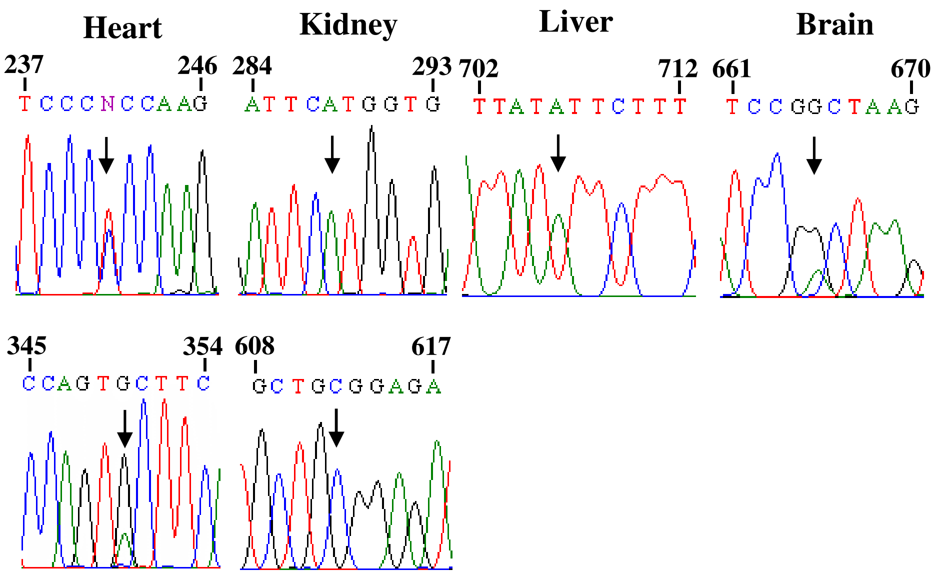

C

Tissues	nucleotide	amino acids
Heart	T 241 T/C	no change
	A 350 G	Thr 99 Ala
Kidney	G 288 A	Arg 78 His
	T 612 C	Val 186 Ala
Liver	G 706 A	outside ORF
Brain	A 665 G	outside ORF
