# Supplementary material for: A Novel Human TPIP Splice-Variant (TPIP-C2) mRNA, Expressed in Human and Mouse Tissues, Strongly Inhibits Cell Growth in HeLa Cells
Source: PLoS One. 2011 Dec 2;6(12):e28433. doi: 10.1371/journal.pone.0028433 (PMC3229583; doi:10.1371/journal.pone.0028433)
Supplement: Table S1 — Homology of TPIP-C2 CDNA with human genome. (DOC) [file pone.0028433.s004.doc]

**Supporting Table 1. Homology of TPIP-C2 CDNA with human genome**

| ***Details of homologous sequence [Accession] (Length of mRNA/ protein)*** | ***E-***  ***value*** | ***Identity*** | *Score bits* | *Homology w.r.t.* ***TPIP-C2*** |
| --- | --- | --- | --- | --- |
| BLASTN 2.2.13 nucleotide sequence | | | | |
| TPIPδ [NM_001141968]  (1833) | 0.0 | 680/682  (99%) | 1247 | 324-1005  *(1042-1722) |
| TPIPγ [NM_199254]  (1974) | 0.0 | 680/682  (99%) | 1247 | 324-1005  *(1183-1863) |
| TPIPα [NM_130785]   (1743) | 0.0 | 680/682  (99%) | 1247 | 324-1005  *(952-1632) |
| TPTEγ  (2085)[NM_199260] | 0.0 | 626/686  (91%) | 918 | 328-1004  *(1284-1960) |
| TPTEβ [NM_199259] (2145) | 0.0 | 626/686  (91%) | 918 | 328-1004  *(1344-2020) |
| TPTEα [NM_199261]   (2199) | 0.0 | 626/686  (91%) | 918 | 328-1004  *(1398-2074) |
| TPIP pseudogene on chromosome 13 [NR_002815.1|] (2727) | 0.0  5e-72  3e-^60^  1e-^48^ | 450/469 (96%)  177/189 (94%)  154/166 (93%)  144/159 (91%) | 756  278  239  200 | 3-468  *(1131-1596)  818-1005  *(2426-2610)  654-818  *(2086-2250)  472-618  *(1928-2086) |
| BLASTP 2.2.13 amino acids sequence | | | | |
| TPIPγ (522) [[NP_954863](http://www.ncbi.nlm.nih.gov/entrez/viewer.fcgi?db=protein&val=40549425)] | 4e-114 | 192/193  (99%) | 408 | 1-193  * (330-522) |
| Transmembrane phosphoinositide 3 phosphatase and tensin homolog 2(482)[[CA73539](http://www.ncbi.nlm.nih.gov/entrez/viewer.fcgi?db=protein&val=40549425)] | 4e-114 | 192/193  (99%) | 408 | 1-193  * (290-482) |
| TPIPδ (411) [[NP_](http://www.ncbi.nlm.nih.gov/entrez/viewer.fcgi?db=protein&val=40549425)001135440] | 1e-113 | 192/193  (99%) | 407 | 1-193  * (219-411) |
| TPIPα (445) [NP_570141] | 1e-113 | 192/193  (99%) | 407 | 1-193  *(253-445) |
| TPTE2 protein (193) [AAI28148] | 4e-112 | 193/193  (100%) | 402 | 1-193  *(1-193) |
| hCG1728574 (199) [EAX08357] | 3e-100 | 177/199 (89%) | 362 | 1-193  *(1-199) |
| TPTEβ (533) [AAH28719] | 7e-88 | 153/193  (80%) | 321 | 1-193  *(330-522) |
| TPTEγ  (513) [AAP45144] | 1e-87 | 153/193  (80%) | 321 | 1-193  *(310-502) |
| TPTEδ (413) [AAP45145] | 2e-87 | 153/193  (80%) | 320 | 1-193  *(210-402) |
| TPTEα (551) [NP_954870] | 1e-86 | 152/193  (78%) | 318 | 1-193  *(348-540) |
| TPIPβ (326)  [ CAD13145] | 3e-57 | 105/105 (100%) | 219 | 1-105  *(199-303) |

**():** Total number of nucleotide/amino acids, **[]**: Accession No., I: Identity, *****: Homology with respect to (w.r.t.) the subject nucleotide/ amino acid sequences
