# Supplementary material for: A Novel Human TPIP Splice-Variant (TPIP-C2) mRNA, Expressed in Human and Mouse Tissues, Strongly Inhibits Cell Growth in HeLa Cells
Source: PLoS One. 2011 Dec 2;6(12):e28433. doi: 10.1371/journal.pone.0028433 (PMC3229583; doi:10.1371/journal.pone.0028433)
Supplement: Table S2 — Presence of TPIP-C2 SINE and LINE in human transcripts. (DOC) [file pone.0028433.s005.doc]

**Supporting Table 2. Presence of TPIP-C2 SINE and LINE in human transcripts**

| **Transcript, nt (Ch)** | **Position** | **Accession no.** | **Score** | **Identity** | **E value** | **Query**  **[Subject]** |
| --- | --- | --- | --- | --- | --- | --- |
| TPIP pseudogene, 2727nt (13) | - | [gi|84872100](http://www.ncbi.nlm.nih.gov/entrez/query.fcgi?cmd=Retrieve&db=Nucleotide&list_uids=84872100&dopt=GenBank) | 509 | 326/335 (97%) | 1e-161 | 7-340  [1135-1467] |
| Fucosyl transferase 2, 3088nt mRNA(19q 13.3) | 3’UTR | [gi|56711329](http://www.ncbi.nlm.nih.gov/entrez/query.fcgi?cmd=Retrieve&db=Nucleotide&list_uids=56711329&dopt=GenBank) | 151  89.7 | 83/91 (91%)  84/97 (86%) | 1e-25  3e-17 | 151-241  [1505-1594] |
| hypothetical protein(LOC642090), 2235 nt (X) | 5’UTR | [gi|89060852](http://www.ncbi.nlm.nih.gov/entrez/query.fcgi?cmd=Retrieve&db=Nucleotide&list_uids=89060852&dopt=GenBank) | 115 | 76/82 (92%) | 6e-251 | 145-241  [2346-2250] |
| zn finger protein 445 (ZNF445), 9105 nt (3p 21.32) | 3’UTR  5’UTR | [gi|56797755](http://www.ncbi.nlm.nih.gov/entrez/query.fcgi?cmd=Retrieve&db=Nucleotide&list_uids=56797755&dopt=GenBank) | 115 | 79/86 (91%)  42/48 (87%) | 6e-23  1e-04 | 151-241  [9020-9105]  151-198  [149-102] |
| choroideremia, transcript variant 1,5442 nt (Xq 21.1) | 3’UTR | [gi|82617643](http://www.ncbi.nlm.nih.gov/entrez/query.fcgi?cmd=Retrieve&db=Nucleotide&list_uids=82617643&dopt=GenBank) | 115 | 76/82 (92%) | 6e-25 | 151-232: SINE  [3439-3520] |
| Homo sapiens ZNF673, 2308nt (Xp 11.3) | 3’UTR | gi|8923321 | 109 | 82/91 (90%) | 3e-23 | 151-241  [1400-1310] |
| Adenylate kinase 1,2271nt(9q 34.1) | 3’UTR | [gi|4502010](http://www.ncbi.nlm.nih.gov/entrez/query.fcgi?cmd=Retrieve&db=Nucleotide&list_uids=4502010&dopt=GenBank) | 107 | 78/86 (90%) | 1e-22 | 156-241  [1568-1653] |
| C22orf25, 2291nt (22q 11.21) | 3’UTR | [gi|38488711](http://www.ncbi.nlm.nih.gov/entrez/query.fcgi?cmd=Retrieve&db=Nucleotide&list_uids=38488711&dopt=GenBank) | 107 | 75/82 (91%) | 1e-22 | 151-232  [1782-1701] |
| FKBP14, 2247nt (7p 15.1) | 3’UTR | [gi|51593093](http://www.ncbi.nlm.nih.gov/entrez/query.fcgi?cmd=Retrieve&db=Nucleotide&list_uids=51593093&dopt=GenBank) | 105 | 62/65 (95%) | 5e-22 | 151-215  [2175-2239] |
| TMF1, 6895nt (3p^21-p12^) | 3’UTR | [gi|110347442](http://www.ncbi.nlm.nih.gov/entrez/query.fcgi?cmd=Retrieve&db=Nucleotide&list_uids=110347442&dopt=GenBank) | 103 | 79/88 (89%) | 2e-21 | 145-232  [4929-5016] |
| TNFAIP8L1, 2209 nt (19p 13.3) | 3’UTR | [gi|22748780](http://www.ncbi.nlm.nih.gov/entrez/query.fcgi?cmd=Retrieve&db=Nucleotide&list_uids=22748780&dopt=GenBank) | 103 | 73/80 (91%) | 2e-21 | 151-230  [1477-1556] |
| C5orf24, 4269nt (5q 31.1) | 3’UTR | [gi|40255110](http://www.ncbi.nlm.nih.gov/entrez/query.fcgi?cmd=Retrieve&db=Nucleotide&list_uids=40255110&dopt=GenBank) | 103 | 70/76 (92%) | 2e-21 | 151-226  [1729-1804] |
| PRPF4, 2756nt (9q31-q33) | 3’UTR | [gi|34222192](http://www.ncbi.nlm.nih.gov/entrez/query.fcgi?cmd=Retrieve&db=Nucleotide&list_uids=34222192&dopt=GenBank) | 101 | 78/87 (89%) | 8e-21 | 146-232  [2095-2181] |
| PCDHB16, 4827nt (5q 31) | 3’UTR | [gi|14195604](http://www.ncbi.nlm.nih.gov/entrez/query.fcgi?cmd=Retrieve&db=Nucleotide&list_uids=14195604&dopt=GenBank) | 101 | 73/79 (92%) | 8e-21 | 151-228  [4097-4019] |
| NDUFV3, transcript variant2, 1056nt (21q 22.3) | 3’UTR | [gi|48255925](http://www.ncbi.nlm.nih.gov/entrez/query.fcgi?cmd=Retrieve&db=Nucleotide&list_uids=48255925&dopt=GenBank) | 101 | 81/91 (89%) | 8e-21 | 151-241  [604-514] |

Query: TPIP-C2 (1-340 nt sequence containing TPIP-C2 LINE and TPIP–C2 SINE), Subject: homologous subject sequence
